# Cysteamine improves growth and the GH/IGF axis in gilthead sea bream (*Sparus aurata*): *in vivo* and *in vitro* approaches

**DOI:** 10.3389/fendo.2023.1211470

**Published:** 2023-07-20

**Authors:** Albert Sánchez-Moya, Sara Balbuena-Pecino, Emilio J. Vélez, Miquel Perelló-Amorós, Irene García-Meilán, Ramón Fontanillas, Josep Àlvar Calduch-Giner, Jaume Pérez-Sánchez, Jaume Fernández-Borràs, Josefina Blasco, Joaquin Gutiérrez

**Affiliations:** ^1^ Department of Cell Biology, Physiology and Immunology, Faculty of Biology, Universitat de Barcelona, Barcelona, Spain; ^2^ Skretting Aquaculture Research Centre, Stavanger, Norway; ^3^ Nutrigenomics and Fish Growth Endocrinology Group, Institute of Aquaculture Torre de la Sal (IATS, Spanish National Research Council (CSIC)), Castellón, Spain

**Keywords:** cysteamine, gilthead sea bream, myocyte, GH, IGF, somatotropic axis, aquaculture, feed additive

## Abstract

Aquaculture is the fastest-growing food production sector and nowadays provides more food than extractive fishing. Studies focused on the understanding of how teleost growth is regulated are essential to improve fish production. Cysteamine (CSH) is a novel feed additive that can improve growth through the modulation of the GH/IGF axis; however, the underlying mechanisms and the interaction between tissues are not well understood. This study aimed to investigate the effects of CSH inclusion in diets at 1.65 g/kg of feed for 9 weeks and 1.65 g/kg or 3.3 g/kg for 9 weeks more, on growth performance and the GH/IGF-1 axis in plasma, liver, stomach, and white muscle in gilthead sea bream (*Sparus aurata*) fingerlings (1.8 ± 0.03 g) and juveniles (14.46 ± 0.68 g). Additionally, the effects of CSH stimulation in primary cultured muscle cells for 4 days on cell viability and GH/IGF axis relative gene expression were evaluated. Results showed that CSH-1.65 improved growth performance by 16% and 26.7% after 9 and 18 weeks, respectively, while CSH-3.3 improved 32.3% after 18 weeks compared to control diet (0 g/kg). However, no significant differences were found between both experimental doses. CSH reduced the plasma levels of GH after 18 weeks and increased the IGF-1 ones after 9 and 18 weeks. Gene expression analysis revealed a significant upregulation of the *ghr-1*, different *igf-1* splice variants, *igf-2* and the downregulation of the *igf-1ra* and *b*, depending on the tissue and dose. Myocytes stimulated with 200 µM of CSH showed higher cell viability and mRNA levels of *ghr1*, *igf-1b*, *igf-2* and *igf-1rb* compared to control (0 µM) in a similar way to white muscle. Overall, CSH improves growth and modulates the GH/IGF-1 axis *in vivo* and *in vitro* toward an anabolic status through different synergic ways, revealing CSH as a feasible candidate to be included in fish feed.

## 1 Introduction

Somatic growth in vertebrates is the result of the positive balance between metabolic and hormonal stimuli, such as the ones promoted by diet and the growth hormone (GH)/insulin-like growth factor (IGF) axis at both systemic and local level, faced to negative regulators, as somatostatin (SS) and myostatins (1–4). GH is a peptide hormone synthetized and released by somatotroph cells located in the adenohypophysis. GH secretion is modulated by a complex system of stimulators and inhibitors composed of more than 30 molecules among peptides, hormones and neurotransmitters. Some of the most important positive regulators are the growth hormone releasing hormone (GHRH), ghrelin and some amino acids, and the negative ones the SS and the IGF feedback system (2, 5–7). The pulsatile nature of GH and IGF secretion on a daily and seasonal basis has been largely established in fish and other higher vertebrates and the frequency and magnitude of secretion can be modulated by some factors as age, environmental conditions, diet, stress, adiposity and exercise, among others etc (4, 7–11). Once the GH is released to plasma, it can bind to GH binding proteins or its ubiquitous distributed receptors (GHR), triggering tissue growth, modulating fuel mobilization and utilization, and promoting the synthesis of downstream molecules (1).

GH has pleiotropic effects and one of the most important is the stimulation of the IGFs (IGF-1 and IGF-2) synthesis and secretion by the liver, the main endocrine IGF source, but also in other tissues including the muscle, in which IGFs exert both paracrine and autocrine functions (1, 2, 4, 12). IGF-1 and IGF-2 are delivered by the IGF binding proteins (IGFBPs), which facilitate their transport, increase their half-life and modulate the IGF actions, depending on the physiological context (1, 13, 14). These IGFs are then recognized by its receptors (IGF-1Ra and IGF-1Rb), initiating the signaling by the mitogen activated protein kinase (MAPK)/ERK and the phosphatidylinositol 3-kinase (PI3K)/AKT pathways (4, 15, 16). IGFs modulate nutrient metabolism and promote cell proliferation and differentiation with a relevant effect on muscular tissue. These mitogenic an anabolic effects result in an improved somatic growth (1, 14, 15, 17).

SS family is an antique but diverse cluster of peptides that presents a key disulfide bond between cysteines, with somatostatin-14 and -28 as its active forms. Furthermore, different studies suggest that other members of this family (prosomatostatins) can play a significant role during zebra fish development (18).It is well known that SS is one of the main inhibitors of GH production and secretion in the adenohypophysis (2, 19). Furthermore, SS reduces the production of IGFs as well as the sensitivity of peripheral tissues to both GH and IGFs by downregulating their corresponding receptors. SS is mainly produced in hypothalamus and gastrointestinal tract, carrying out an important role in systemic and local control of growth. This facilitates the coordination with other processes such as development, metabolism and reproduction, resulting in a harmonic growth (19–21).

Cysteamine (2-Aminoethane-1-thiol; CSH) is the simplest aminothiol endogenously synthesized by animal cells during the cysteine and coenzyme A degradation, being rapidly metabolized and excreted by the organism (22, 23). CSH is the biosynthetic precursor of hypotaurine, which is rapidly converted to taurine, a semi-essential amino acid particularly important in carnivore nutrition (24–26). In addition, CSH promotes the transport of L-cysteine into cells, which will be used, among others, to synthesize glutathione, the main endogenous antioxidant of the organism. CSH has commonly been administered as a salt form (i.e., as hydrochloride and bitartrate) for therapeutic purposes including the treatment of cystinosis, skin lightener and as a radioprotective agent. Additionally, CSH has been used in higher doses to provoke digestive ulcers for animal research purposes, since it is capable to increase the secretion of gastrin, gastric acid and ghrelin, and the reduction of angiogenesis, enabling ulceration instead of wound healing (23, 27–29). Its properties are mainly due to its thiol group, which can act as an antioxidant and reducing agent, breaking up the disulfide bounds as it occurs between cysteines (22, 23, 27).

Some studies demonstrated that CSH drops SS in different tissues, being suggested the disulfide bond-breaking mechanism as the responsible of its depletion, although this model is not well established yet (30, 31). Furthermore, it was observed that the *in vivo* administration of CSH increased the levels of GH, IGF-1 and their receptors in different tissues in mammals and fish. The synergic effect of this SS reduction and the improvement of the GH/IGF axis is reflected in a boosted growth in several species (32–34). Nevertheless, there have also been reported some negative effects in endocrine control of growth in CSH treated groups in a dose-dependent and temporal manner, with a reduction of GHRH and depression of the GH/IGF system in rat, swine and sheep (8, 35–38). In this sense, it has been suggested that the increased levels of plasmatic GH associated to moderate CSH doses, are consistent with SS depletion, but the mechanism why GH secretion is disrupted with higher CSH doses is not well-understood (8, 37). Many of the previous studies in vertebrates were made using *in vivo* models, where most of the parameters studied were globally influenced by the interaction among tissues. Nevertheless, the information about the local and specific effects of CSH, like those that we could study *in vitro*, remains scarce. CSH has been supplemented in cell media for mammalian oocyte maturation (39), but the effects of CSH on muscle cells is still unknown. CSH has been proposed as a growth promoter additive in animal nutrition, including sheep, chicken, yak and pig (38, 40, 41) and recently, also in different fish species, such as in red tilapia (*Oreochromis niloticus*), orange-spotted grouper (*Epinephelus coioides*) and common carp (*Cyprinus carpio*) (42–44). However, there is not a consensus about the underlying mechanisms and the optimal inclusion dose, due to its association with several negative effects on growth, hormonal regulation, oxidative stress and welfare (27, 44–46).

Overall, it is suggested that CSH could be a useful additive in animal nutrition since it could improve growth through diverse and synergic mechanisms. Nevertheless, it is necessary to determine the most efficient CSH inclusion level in diet considering the physiological characteristics of each species and the growth stage, as to the apparition of adverse effects could be close to the ideal dosage.

In this context, the objective of this study was to investigate for the first time in gilthead sea bream (*Sparus aurata*), one of the most important fish in Mediterranean aquaculture, the effects of an *in vivo* and *in vitro* CSH supplementation at different doses on somatic growth, the GH/IGF axis and myocytes viability.

## 2 Materials and methods

### 2.1 Experimental diets

The diets used in this trial were based on a practical commercial diet for gilthead sea bream at these stages of growth. Five experimental diets were formulated and produced by Skretting Aquaculture Research Center (Skretting-ARC, Stavanger, Norway), by an extrusion process with a 30% of fish meal and 9% of fish oil to fulfill the essential nutritional requirements. In detail, for the Phase 1 of the trial, two diets of 1 mm pellet size were used: an unsupplemented one as control diet (Control), and the same one but with the addition of 1.65 g of cysteamine hydrochloride (CSH; Ref. 30080-100G; Sigma-Aldrich, Tres Cantos, Madrid) per Kg of feed (CSH-1.65). The diets for the Phase 2 were the same Control and CSH-1.65 diets used in the Phase 1 but with the pellet size adjusted for bigger fish (1.8 mm). Furthermore, a new experimental group was added in Phase 2, with the CSH dose doubled at 3.3 g CSH/Kg of feed (CSH-3.3). All the diets within the same phase were isolipidic and isonitrogenous. The detailed formulation of diets is shown in **Table 1**.

**Table 1:** Ingredients and proximal composition of the diets.

	Phase 1 (1 mm)		Phase 2 (1.8 mm)		
	Control	CSH-1.65	Control	CSH-1.65	CSH-3.3
Ingredient (%)					
Wheat	19.89		24.89		
Corn gluten	9		6		
Wheat gluten	9.4		7.13		
Soya concentrate	20		20		
Fish meal	30		30		
Fish oil	9.01		9.31		
DL-Methionine	0.17		0.2		
Phosphate	1.52		1.69		
Antioxidant	0.03		0.03		
Vitamin premix	0.02		0.02		
Cysteamine HCl	0	0.165	0	0.165	0.33
Proximal composition (%)					
Dry matter (DM)	91.99		91.77		
Moisture	8.01		8.24		
Protein (% DM)	48		45		
Lipid (% DM)	15		15		
Starch (% DM)	7.49		7.53		
Ash (% DM)	12.98		15.33		

### 2.2 Animals and ethic statement

Approximately one thousand gilthead sea bream fingerlings provided by Piscimar (Burriana, Spain) with 1.8 ± 0.3 g of body weight, were randomly distributed in six circular tanks of 200 L and three tanks of 400 L at the same initial biomass density of 0.75 g fish/L, and maintained in the fish facilities of the Scientific and Technological Centers, Faculty of Biology, University of Barcelona. The tanks are part of a semi-closed recirculation system with a constant 36‰ salinity, 22 ± 1 °C and under a 13 h light/11 h dark photoperiod.

For the Phase 1, the fish of two 200 L tanks and one 400 L were fed with the control diet (Control, n = 3) and four 200 L tanks and two 400 L with the experimental diet (CSH-1.65, n = 6) for nine weeks. According to fish requirements, the fish were fed with a daily 3.5% ration distributed in four meals per day during the first five weeks, and the next four weeks at 3% ration allocated in three meals per day.

Once the Phase 1 ended and the corresponding biometric data and samples were obtained as detailed below, the remaining fish were recovered and returned to their respective tanks maintaining similar biomass density to later start Phase 2. Pellet composition and size were slightly adjusted for this Phase 2 (**Table 1**) and fish were adapted to the new feed mixing them 50/50 for five days. The half of the tanks that were fed with the CSH-1.65 diet in Phase 1 had their CSH dose doubled with the new CSH-3.3 diet, whereas Control and the half of CSH-1.65 tanks remained in the same diet that in the Phase 1. The feeding ration was set at 2.5% the first three weeks, 2% the following three weeks and, finally, 1.75% de last two weeks, distributed in three meals per day. Hence, each condition for the Phase 2 (Control, CSH-1.65 and CSH-3.3) had two 200 L and one 400 L tanks (n = 3).

### 2.3 Biometric parameters and sampling

At the end of the Phase 1 and Phase 2 all fish were fasted overnight and properly anesthetized with MS-222 (100 mg/L) (Sigma-Aldrich) before being measured and weighed. The technical replicate for biometric data and indexes was the mean of the tank. Besides, three to five samples/tank were taken from the caudal vein for hormone analysis. Then, twenty fish of each 200 L tank and forty fish of 400 L tank were sacrificed by anesthetic overdose (300 mg/L), confirmed by decapitation, and the tissues were extracted and weighted for the calculation of somatic indexes: Specific Growth Rate (SGR) = [ln (final body weight) – ln (initial body weight)] * (days)^-1^ * 100; Condition Factor (CF) = (body weight/body length^3^) * 100; Viscerosomatic Index (VSI) = (viscera weight/body weight) * 100; Hepatosomatic Index (HSI) = (liver weight/body weight) * 100; Mesenteric Fat Index (MFI) = (mesenteric fat weight/body weight) * 100. For the relative gene expression analyses of the Phase 2, fourteen fish of each condition (four to six samples/tank) were sampled and the white muscle, liver and stomach were immediately frozen in liquid nitrogen and stored at -80°C until further analysis.

### 2.4 Plasma GH and IGF-1

GH concentration in plasma was assayed by a homologous gilthead sea bream radioimmunoassay in accordance with Martínez-Barberá et al. (1995) (44). The sensitivity and midrange (ED50) of the assay were 0.15 and 1.8 ng/ml, respectively. Plasma IGFs were extracted by the acid–ethanol cryoprecipitation (45), and the IGF-1 concentration was measured by means of a generic fish IGF-1 RIA validated for the Mediterranean perciform fish (46). The sensitivity and mid-range of the assay were 0.05 and 0.7–0.8 ng/mL, respectively.

### 2.5 Primary culture of myocytes and experimental treatments

Seven independent white muscle satellite cell cultures were performed following the protocol previously described by Montserrat et al. (2007) (47). Briefly, around 40 juvenile fish (5 to 15 g/fish) supplied by local hatchery were used for each cell culture. The fish were sacrificed by a blow to the head and their external surfaces were sterilized. Then, fish were dissected and the epaxial white muscle tissue was collected in cold buffered Dulbecco’s Modified Eagle’s Medium (DMEM), containing 1% (v/v) antibiotic/antimycotic solution and supplemented with 15% (v/v) horse serum (HS). Subsequently, muscle was minced to small fragments and centrifuged (3000 rcf, 5 min), washed and enzymatically digested with 0.2% collagenase type IA. The obtained suspension was centrifuged and the pellet washed, resuspended, triturated by repeated pipetting and centrifuged. After that, the tissue fragments were digested twice with 0.1% trypsin solution prepared in DMEM and gentle agitation. After each digestion the remained fragments were pelleted (300 rcf, 1 min) and diluted in complete medium (DMEM supplemented with 15% of HS) to block trypsin activity. Then, the supernatant was centrifuged (300 rcf, 20 min) and the obtained pellet resuspended, forced to trituration by pipetting and then, the suspension was filtered first on a 100 μm, and subsequently on a 40 μm nylon cell strainer, and finally centrifuged one last time (300 rcf, 20 min). After that, the obtained cells were diluted in growth media (DMEM supplemented with 10% fetal bovine serum and 1% of antibiotic-antimycotic solution) and seeded at a final density of 2105 cells/cm^2^ in poly-L-lysine and laminin precoated 6-well plates (9.6 cm^2^/well) for gene expression or 12-well plates (2.55 cm^2^/well) for the viability assay. Cells were incubated at 23°C and 2.5% CO_2_ in growth medium and medium was changed every 2 days.

CSH (Ref. 30080-100G; Sigma-Aldrich) was diluted in culture medium at doses of 50, 200, 400 and 800 µM and applied at day 4 for 96 h (until day 8 of cell culture development) to determine cell viability (n = 7) and select the most appropriate one. Once the dose of 200 µM was selected, myocytes at day 4 were incubated with CSH at 200 µM for 96 h to evaluate gene expression (n = 7). These days were chosen due to cells retain the ability to proliferate but also have the capacity to start fusing and differentiating (47). This is supported by data reported in previous publications (15, 47–51). In the control group, the cells were not incubated with CSH.

### 2.6 Cell viability assay

The methylthiazolyldiphenyl-tetrazolium bromide (MTT) assay was used to assess cell viability as explained before (47). Briefly, after CSH exposure, cells were washed twice and incubated for 5 h in DMEM with a final concentration of 0.5 mg/mL of MTT (M5655, Sigma-Aldrich). Cells were washed with PBS and the blue formazan crystals were allowed to resuspend in DMSO. The viability values were obtained from the absorbance measured at 570 nm in duplicate 96-wells, with correction at 650 nm, using a microplate reader (Tecan Infinite M200, Männedorf, Switzerland). The value from cells containing PBS instead of MTT was also subtracted. Data are presented as a fold change relative to the control group (0 µM) of each independent cell culture (n = 7 independent cultures).

### 2.7 RNA extraction, cDNA synthesis and qPCR analysis

Total RNA was extracted from stomach (~30 mg), liver (~30 mg) and white muscle (~100 mg) (n = 14) homogenates as described in Sánchez-Moya et al. (2022) (52), or from cell samples collected in triplicate wells of a 6-well plates at day 8 with 1 ml of TRI Reagent® Solution (Applied Biosystems, Alcobendas, Spain). In the case of the *in vivo* samples, Precellys® Evolution Homogenizer cooled at 4°C with Cryolys® (Bertin Technologies, Montigny-le–Bretonneux, France) was used. RNA extraction and purification was conducted following the manufacturer’s recommendations. RNA concentration and purity was determined using the NanoDrop 2000 (ThermoScientific, Alcobendas, Spain). RNA integrity was verified in a 1% agarose gel stained with 3% SYBR Safe DNA gel stain (ThermoScientific) observing the banding pattern of 28S:18S ribosomal RNA. Previously to reverse transcription, samples were treated with DNase I (Life Technologies, Alcobendas, Spain) following the manufacturer’s recommendations to remove any traces of genomic DNA. Lastly, cDNA synthesis was carried out from 1 µg of RNA with the Transcriptor First Strand cDNA Synthesis Kit (Roche, Sant Cugat del Vallés, Spain), using random hexamer primers and anchored oligo(dT)15.

Genes chosen for gene expression analysis were key components of the GH/IGF axis, namely GHRs (*ghr-1, ghr-2*), IGFs (*igf-1a, igf-1b, igf-1c, igf-2*), IGF receptors (*igf-1ra, igf-1rb*) and IGFBPs (*igfbp-1a, igfbp-2a, igfbp-4*). Relative mRNA expression analyses were performed via qPCR from the cDNA samples, following the MIQUE’s guidelines in Hard-Shell® 384-well PCR plates and a CFX384TM Real-Time System (Bio-Rad, El Prat de Llobregat, Spain). The analyses were performed in triplicate using 2.5 μL of the iTaq Universal SYBR Green Supermix (Bio-Rad), 250 nM of both the forward and reverse primers, and 1 μL of diluted cDNA for each sample made up to a final volume of 5 μL. The reactions consisted of an initial denaturation step of 3 min at 95°C, 40 cycles of 10 s at 95°C, 30 s at 60–69°C (primer dependent) followed by an amplicon dissociation analysis from 55 to 95°C at a 0.5°C increase every 30 s. The sequences, melting temperatures and GenBank accession numbers of the primers used in the Real-Time quantitative PCR analysis are displayed in **Supplementary Table S1**. The mRNA expression of each target gene was calculated relative to the geometric mean of the two most stable reference genes from the four that were determined for each of the tissues and the *in vitro* samples according to the geNorm algorithm implemented in the Bio-Rad CFX Manager v. 3.1. software. The different housekeeping genes tested were ribosomal protein S18 (*rps18*), elongation factor 1 alpha (*ef1α*), beta-actin (*β-actin*) and ribosomal protein L27 (*rpl27*).

### 2.8 Statistical analyses

Statistical analyses were performed with IBM SPSS Statistics v.22 (IBM, Armonk, USA) software whilst all the figures were prepared with GraphPad Prism v.7 (GraphPad Software, La Jolla California USA, www.graphpad.com). Previous to statistical comparison among treatments, all data was tested for normality and homoscedasticity by Shapiro-Wilk and Levene’s tests, respectively, and the identification of outliers was assessed by IQR’s. When the groups compared were two, as in the Phase 1 of the *in vivo* trial and the *in vitro* gene expression analyses, a Student’s t-test was done. When there were three or more groups, as in Phase 2, tissue relative expression and MTT assay, a one-way analysis of variance (one-way ANOVA) followed by Tukey’s *post hoc* test were carried out. Data is presented as mean ± S.E.M. and statistical differences were considered significant when p-value < 0.05.

## 3 Results

### 3.1 Growth and hormone analysis

Growth performance and somatic indexes are shown in **Table 2**. Fish fed with CSH presented larger final body weight compared to the Control group regardless of the phase and dose. The CSH-1.65 group weighed +16% after Phase 1 and +26.7% after Phase 2, compared to the Control group respectively. The highest weight gain was found in the CSH-3.3 fed group, with a +32.2% of increment respect to the Control fish. Total length also increased in proportion to weight in all the CSH fed fish, which resulted in no differences for CF. SGR was higher in Phase 1 for CSH-1.65 compared to Control; but in Phase 2, even though this group presented a higher value (2.02 vs 2.15), the differences were not significant. On the other hand, CSH-3.3 got the highest value (2.29), which was significantly different to that of Control group. No differences were found for VSI and HSI after Phase 1 and 2. MFI were increased after Phase 2 in CSH-fed groups in a dose-dependent manner, showing a proportionally higher fat deposition in fish fed with CSH-3.3 diet.

**Table 2:** Growth performance, somatic indexes and hormone levels of gilthead sea bream juveniles (*Sparus aurata*) supplemented with cysteamine hydrochloride after 9 and 18 weeks.

	Phase 1 (week 1 to 9)		Phase 2 (week 10 to 18)		
	Control	CSH-1.65	Control	CSH-1.65	CSH-3.3
	*n = 3*	*n = 6*	*n = 3*	*n = 3*	*n = 3*
**IBW (g)**	1.77 ± 0.03	1.81 ± 0.03	13.13 ± 0.4 **b**	15.4 ± 0.4 **a**	14.85 ± 0.3 **a**
**IBL (cm)**	4.56 ± 0.04	4.63 ± 0.03	8.23 ± 0.07 **b**	8.6 ± 0.02 **ab**	8.47 ± 0.08 **a**
**FBW (g)**	12.84 ± 0.25	14.9 ± 0.1***	41.4 ± 0.5 **b**	52.45 ± 1 **a**	54.74 ± 1 **a**
**BL (cm)**	8.17 ± 0.07	8.5 ± 0.03***	11.7 ± 0.04 **b**	12.65 ± 0.04 **a**	12.55 ± 0.06 **a**
**CF^1^**	2.34 ± 0.03	2.39 ± 0.02	2.57 ± 0.03	2.61 ± 0.08	2.75 ± 0.03
**SGR^2^**	3.25 ± 0.05	3.46 ± 0.03**	2.015 ± 0.04 **b**	2.15 ± 0.04 **ab**	2.29 ± 0.07 **a**
**VSI^3^**	7.89 ± 0.1	7.76 ± 0.06	6.03 ± 0.31	5.76 ± 0.1	6.18 ± 0.14
**HSI^4^**	1.94 ± 0.12	1.91 ± 0.03	1.37 ± 0.05	1.29 ± 0.08	1.47 ± 0.04
**MFI^5^**	1.04 ± 0.13	1.05 ± 0.06	0.81 ± 0.03 **b**	0.99 ± 0.05 **ab**	1.11 ± 0.08 **a**
	*n = 10*	*n = 12-13*	*n = 13*	*n = 13*	*n = 14*
**GH (ng/ml)**	1.72 ± 0.24	1.84 ± 0.23	1.66 ± 0.44 **a**	0.96 ± 0.28 **ab**	0.36 ± 0.14 **b**
**IGF-1 (ng/ml)**	13.83 ± 2.01	22.53 ± 3.23*	16.88 ± 1.72 **a**	23.37 ± 1.96 **b**	21.71 ± 1.7 **ab**

IBW, initial body weight; IBL, initial body length; FBW, final body weight; BL, body length; GH, growth hormone; IGF-1, insulin-like growth factor 1; ^1^Condition Factor (CF) = (body weight/body length^3^) * 100; ^2^Specific Growth Rate (SGR) = [ln (FBW) – ln (IBW)] * (days)^-1^ * 100; ^3^Viscerosomatic Index (VSI) = (viscera weight/body weight) * 100; ^4^Hepatosomatic Index (HSI) = (liver weight/body weight) * 100; ^5^Mesenteric Fat Index (MFI) = (mesenteric fat weight/body weight) * 100.

Significant differences were evaluated by a t-test (p-value: **<0.01; ***<0.001) for Phase 1 and one-way ANOVA followed by a Tukey’s post hoc test for Phase 2. Different letters in the same raw indicate significant differences between groups.

Data are shown as mean ± S.E.M. Tank was used as a technical replicate for the biometric data.

The plasmatic levels of GH and IGF-1 were affected by diet and are shown in **Table 2**. GH concentration did not show differences between the Control and the CSH-1.65 group in Phase 1. However, in Phase 2, GH levels were reduced in fish fed with CSH in a dose-dependent manner. Contrarily to GH, IGF-1 levels were increased significantly in Phase 1 and Phase 2 in those groups fed with CSH-1.65.

### 3.2 qPCR in white muscle, liver and stomach

**Figure 1:**
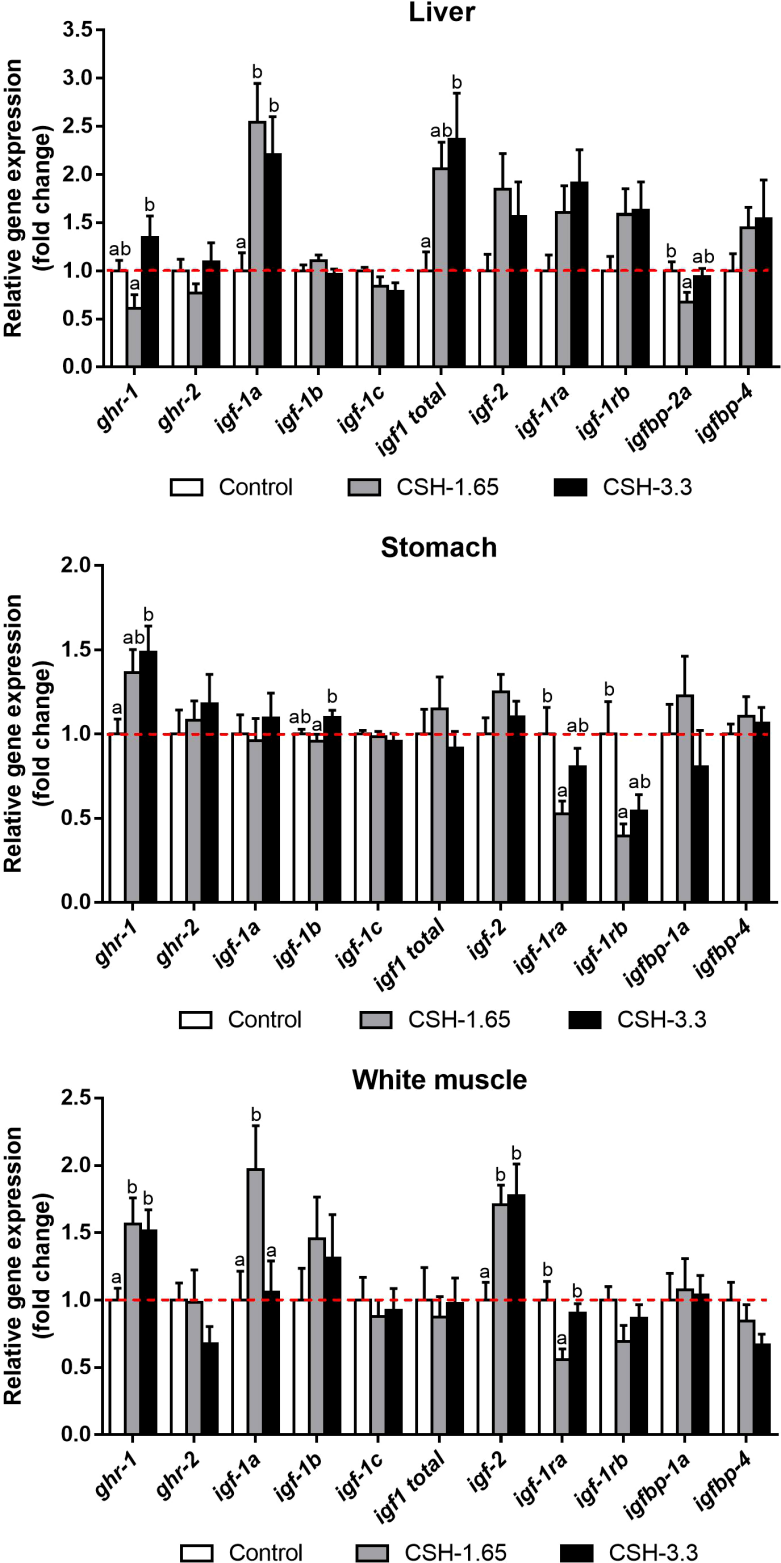
Relative gene expression in liver, stomach and white muscle of gilthead sea bream (*Sparus aurata*) supplemented with CSH at the end of the entire trial (Phase 1 followed by Phase 2). Data are shown as mean ± S.E.M. relativized to Control mean (n = 14). Significant differences between treatments were determinedby one-way ANOVA and Tukey’s *post-hoc* test and are indicated with different letters (p < 0.05).

Relative gene expression in liver, stomach and white muscle at the end of Phase 2 are represented in **Figure 1**. In liver, *ghr-1* expression was slightly reduced and increased in CSH-1.65 and CSH-3.3, respectively, compared to control, showing significant differences between both treated groups. Regarding to *igf-1* expression, *igf-1a* was increased in both doses and the expression of total *igf-1* was only significantly raised at 3.3 dose. Moreover, the binding protein *igfbp-2a* was reduced in CSH-1.65 group compared to control, whereas CSH-3.3 had an intermediate value. No differences were found for *ghr-2, igf-1b, igf-1c, igf-2, igf-1ra, igf-1rb* and *igfbp-4*.

Concerning stomach, CSH supplementation significantly enhanced the transcript levels of *ghr-1* in CSH-3.3 group in a similar manner to white muscle. *Igf-1b* presented significant differences between the treated groups, with a reduction for the CSH-1.65 respect to the CSH-3.3. On the other hand, the expression of the receptors *igf-1ra* and *igf-1rb* were lessened, but only significantly, for the CSH-1.65 diet compared to control. No changes were observed for *ghr-2, igf-1a, igf-1c*, total *igf-1, igf-2, igfbp-1a* and *igfbp-4*.

Relative gene expression in white muscle was also modulated by CSH supplementation in diet. The mRNA levels of the receptor *ghr-1* and the *igf-2* were increased in both groups fed with CSH respect to the Control group. Interestingly, the igf-1 splice variant *igf-1a* doubled its value in the fish fed with the CSH-1.65 dose but not with the CSH-3.3 one, compared to Control. Contrarily to this profile, the *igf-1ra* was downregulated for the CSH-1.65 whereas CSH-3.3 did not change. There were no differences for *ghr-2, igf-1b, igf-1c*, total *igf-1, igf-1rb, igfbp-1a* and *igfbp-4* among groups.

### 3.3 Cell viability

**Figure 2:**
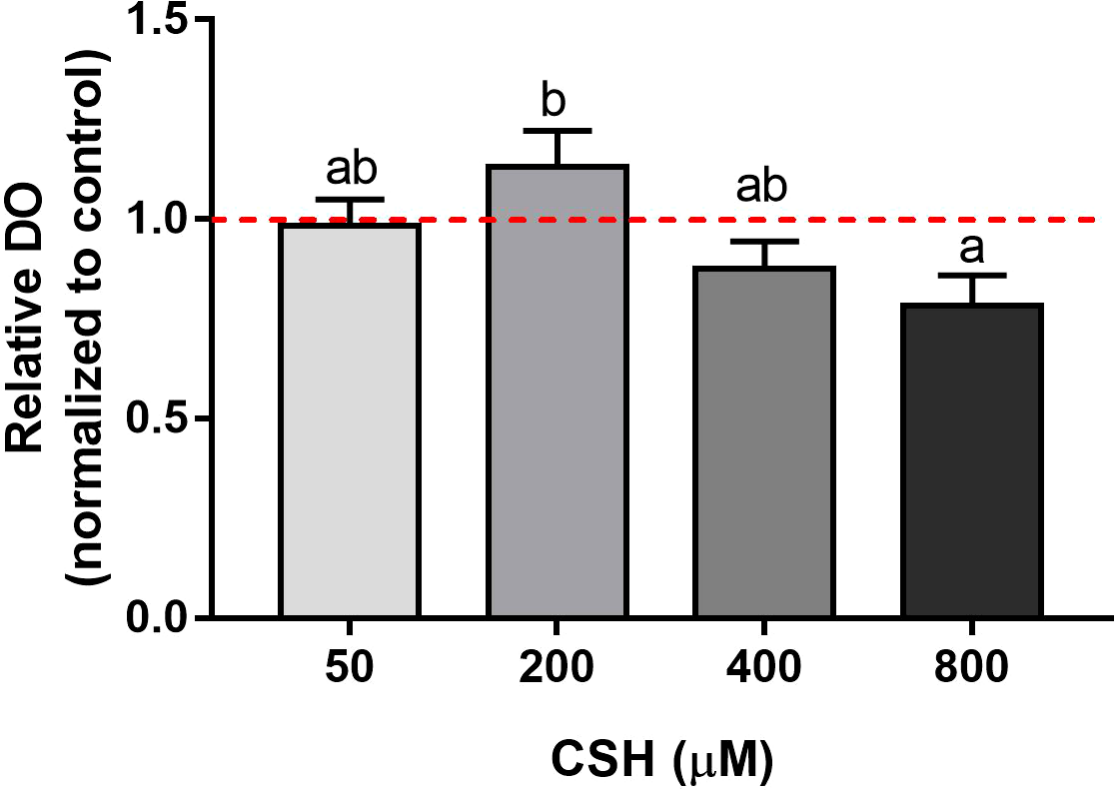
Viability of gilthead sea bream (*Sparus aurata*) myocytes exposed to different concentrations of CSH (50, 200, 400 and 800 µM) and quantified by methylthiazolyldiphenyl-tetrazolium bromide (MTT) assay. Data are shown as fold change of the mean + S.E.M. (n = 7) with respect to the Control group (0 µM, dotted line) for each cell culture. Significant differences between treatments were determined by one-way ANOVA followed by a Tukey’s *post hoc* test and are indicated with different letters (*p* < 0.05).

MTT reduction capacity, as indicator of cell viability, of myocytes exposed to increasing CSH concentrations is shown in **Figure 2**. The CSH 200 µM concentration obtained the highest viability relative value; however, higher doses gradually decreased cell viability until complete cell death occurs at 2 mM (data not shown).

### 3.4 qPCR in myocytes

**Figure 3:**
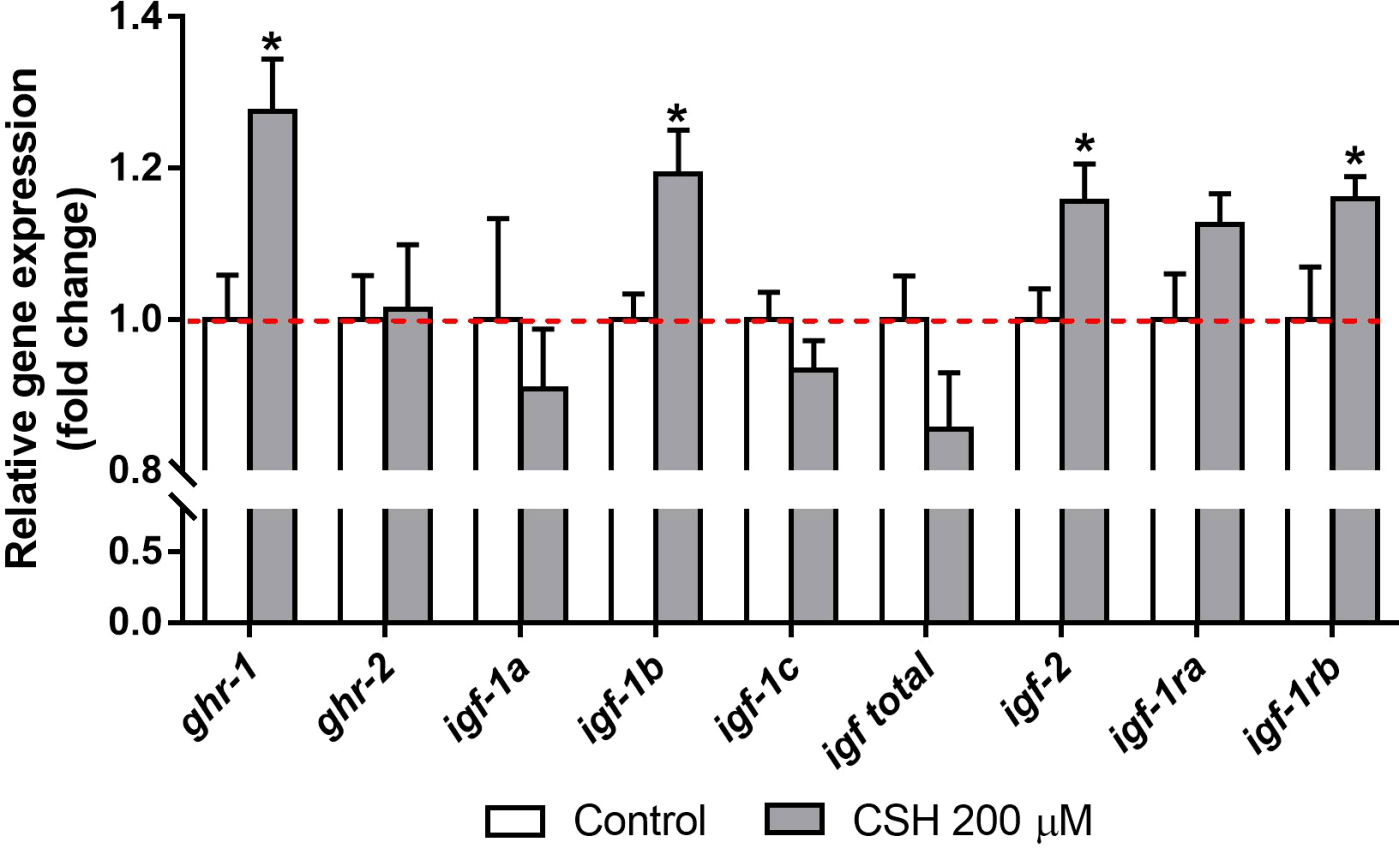
Relative gene expression of gilthead sea bream (*Sparus aurata*) myocytes exposed to CSH (200 µM) from day 4 to day 8 of cell culture development, presented as the fold change with respect to the Control group mean (dotted line). Data are shown as mean + S.E.M. (n = 7). Significant differences between treatments were determined by a *t*-test, and are indicated by asterisks (*p* < 0.05).

Relative gene expression of GH/IGF system in primary cultured myocytes exposed to CSH 200 µM from day 4 to day 8 of cell culture is shown in **Figure 3**. In presence of CSH, the *ghr-1*, the splice variant *igf-1b*, the *igf-2* as well as *igf-1rb* were upregulated compared to the Control group. Nevertheless, no differences were found for *ghr-2, igf-1a, igf-1c*, total *igf-1* and *igf-1ra*.

## 4 Discussion

Feed is the main expenditure in fish production and its cost is being raised due to the increase of raw material price. This, together with the general upward trend to substitute fish meal for more sustainable alternatives including vegetable, insect and waste-derived ingredients, has entailed to increase the research for novel feedstuff without compromising fish growth, quality and competitiveness (53). The use of natural and functional additives has particular interest due to its reduced inclusion level in fish feeds and their attributes as immunostimulants, antioxidants, stress reducers, digestion facilitators and growth promoters (54–57). In this context, CSH appears as a potential candidate to become a food additive to optimize the production of aquaculture species given that CSH has been proved as a growth promoter in some terrestrial vertebrates, and to a lesser extent, in fish (e.g., common carp, red tilapia and orange-spotted grouper) (42–44, 58, 59). Notwithstanding, CSH has also been associated to detrimental effects including digestive ulcers, oxidative stress, reduction of growth and hormonal downregulation of the GH/IGF axis, making the inclusion range narrow (8, 27, 33, 37, 44). In the present work we have demonstrated that the inclusion of 1.65 g of CSH/Kg of feed improved the growth by + 16.1% in 9 weeks (Phase 1) and + 26.7% after 18 weeks (Phase 2) in gilthead sea bream, without modifying somatic indexes. On the other hand, when fish ate the doubled CSH-dose feed (3.3 g/Kg) some differences appeared, as a slightly increased body weight (+ 32.2%), and significantly higher SGR and MFI compared to Control. Therefore, an increasing dose of CSH boosted growth and visceral fat deposition but, at the same time, the low one seemed to have a better relation cost/effect in 9 weeks of trial (Phase 2). It has to be pointed that those differences could be augmented with prolonged periods or higher doses. In line with the obtained results, Li et al. (2013) (44) found that the best CSH inclusion in feed for orange-spotted grouper in an eight weeks trial was 3 g/Kg, with significant lower growth for the 1, 2 and the 4 g/Kg doses, although all of them induced greater growth compared to the control group. This indicated that the optimal point in this species was between the 3 and 4 g/Kg doses. Tse et al. (2009) (43) found similar results on common carp with increased growth with 1, 2 and 3 g/Kg doses. Gonzalez-Plasus et al. (2019) (46) additionally showed marked detrimental effects as reduced growth, deformities and mortality at a dose of 10 g/Kg in common carp. On the other hand, Wardani et al. (2020) (34) found that the optimal dose for tilapia was 0.59 g/Kg. Overall, there is not a consensus about the optimal and toxic CSH dosages in fish, which seem to be species-specific and, consequently, it would depend on their main nutritional requirements and their digestive tract physiology. Thus, as CSH effects are mainly dose-dependent, this highlights the need of fine-tuning CSH inclusion in diets.

The mechanism by which the CSH enhances growth has not been fully understood, although it is generally accepted that CSH effects are originated at the SS-GH-IGF axis level, the main hormonal mechanism responsible of somatic growth in vertebrates (2, 14, 60). It has been largely demonstrated that CSH injection or ingestion is associated with the reduction of SS, probably as consequence of the breakdown of the disulfide bond of SS by the thiol group of CSH, although this process is not completely elucidated (23, 30, 31, 61). This is a key point considering that SS is produced in the hypothalamus but also along the digestive system, and its depletion can result in multiple local effects, as the inhibition of the releasing of different endocrine and paracrine hormones, altering several physiological processes (62). Dohil et al. (2006, 2014) (63, 64) demonstrated that CSH bitartrate is almost completely absorbed in small intestine, but it also has effects in the previous pass through the stomach. Furthermore, its absorption and metabolization in mammals takes place in hours (23, 65). A reduction of SS would trigger the secretion of GH by the adenohypophysis gland as it was previously observed in the literature, where the administration of CSH by different ways improved growth and increased the GH and IGF-1 levels (8, 32, 41, 59). Regarding this, Hu et al. (2016) (41) found a positive correlation between GHRH, GH and IGF-1 levels in fish fed with CSH. Interestingly, and contrary to what most bibliography supports, we report here a CSH dose-dependent reduction of plasmatic GH levels compared to the control group in the Phase 2 of the trial, while IGF-1 levels were significantly increased by CSH, but only significant for the CSH-1.65 group. McLeod et al. (1995a, 1995b) (8, 66) and McElwain et al. (1999) (37) also observed a reduction of the GH levels and modifications in the amplitude and duration of GH release depending on the CSH dose administered. Those authors explained the GH decrement by the previous low levels of GHRH, which would be due to a reduction of catecholamine synthesis in the hypothalamus caused by CSH. Other explanation could be based on the negative feedback regulation of the GHRH/GH/IGF axis. In either case, circulating IGF-1 levels were upraised and the IGF-1/GH ratio was increased in both phases, indicating an ongoing anabolic condition in agreement with the biometric data (67, 68). The increase of the MFI with the high dose could be explained, on the one hand, by the anabolic condition given by the concomitant high IGF-1, promoting adipocyte proliferation and differentiation and better nutrients uptake to the cell, and on the other hand, by the reduction of GH, which has lipolytic effects and plays an important role on energy management (17, 69, 70). The improvement of the IGF-1/GH ratio trough the time could be related with the better response to CSH with an adaptation period of 4-6 weeks and the increasing doses (64). Regarding this, during the design of the present study, it was proposed to double the dose of CSH during the Phase 2, instead of reducing it, since both the GH and IGF-1 are gradually reduced with age (71), and the intensity of the stimulus needed for increasing their levels would be higher.

Generally, the gene expression results of the GH/IGF axis in the *in vivo* model showed two clear patterns in the different tissues. On one hand, an increase in the mRNA levels of the analyzed genes by both CSH doses (1.65 and 3.3) compared to control group (e.g., *ghr-1* and *igf-2* in white muscle). On the other hand, an increase or decrease of gene expression for the low CSH dose (1.65) but in a lesser extent for the 3.3-group, which presented similar values to control group (e.g., *igf-1a* and *igf1-ra* in white muscle). GH and IGFs are recognized by their corresponding receptors (GHR and IGFR, respectively) widely distributed through the different tissues. Two different GHR (GHR-1 and GHR-2) have been described in several fish species, including gilthead sea bream (72, 73). The functional divergence of these two paralogs have not been fully elucidated yet, though it seems that GHR-1 is upregulated under an anabolic status whereas GHR-2 is positively related with stress and energy mobilization signals (1, 5, 44, 60, 73–75). This would be in accordance with the improved growth that we observed in the fish fed with the CSH diets and the upregulation of the *ghr-1* but not the *ghr-2* in white muscle and stomach and interestingly, also in myocyte cell culture. It appears, thereby, that the *in vivo* growth-promoting action of CSH would be partially mediated by the GHR-1, which increase could be a compensatory mechanism in response to the lower circulating GH levels.

IGFs play a key role in skeletal muscle growth and differentiation through the endocrine action of IGF secreted from liver and its own paracrine function. In our results, we found that the fish fed with CSH presented an increase in the *igf-1a* splice variant for both doses and in total *igf-1* for CSH-3.3 in the liver. However, we did not observe differences in *igf-1c* transcript levels among groups, the principal isoform in liver (76). The overexpression of the total *igf-1* observed in liver would explain the increased levels of the IGF-1 showed in plasma (14, 17, 34). Tse et al. (2006) (43) found and exponential increase of the hepatic *igf-1* gene expression and, especially, the *igf-2* in carps fed with CSH (0, 1, 2 and 3 g/Kg) at day 7. However, at day 63 the *igf-1* levels were equalized among treatments compared to control except for the 3 g/Kg dose, suggesting that this dose would continue to stimulate the IGF synthesis. Regarding muscle, Tse et al. (2006) (43) only observed differences at day 63 and for the 3 g/Kg dose. In our results the *igf-1a* was also upregulated in the CSH-1.65 group but not in the CSH-3.3 compared to Control. This could be due to a previous peak of the *igf-1a* in CSH-3.3 group that in the moment of the sampling was in a downregulation step. Integrating the liver and the muscle responses to CSH, these suggest a key point between the 1.65 and 3 g/Kg dose that triggers some anabolic signals. It is interesting to compare the CSH effects on *igf-2* expression in different tissues, emphasizing the significant upregulation responses in muscle and *in vitro* myocytes, but not in liver or stomach. This agrees with the important role of IGF-2 in myocytes proliferation and myogenesis (48, 77) and its overexpression found in muscle of fast-growing catfish family (78) pointing out the role of IGF2 in the muscle of these fish species.

Concerning the *in vitro* experiment, the use of CSH on myocytes had not been studied yet and there is scarce information about CSH effects and doses on other cell type cultures. Besides, the data of this study represents the first approach to understand its direct effects on primary fish myocytes. Beyond our study, CSH has been previously used as antioxidant and maturation-promoter in mammalian oocytes in a range between 25 to 400 µM (39). Here we reported that the maximum non-toxic dose of CSH in gilthead sea bream myocytes under the differentiation phase (day 4 to day 8) is 200 µM, which agrees with these previous works. Regarding the somatotropic axis-related genes, myocytes stimulated with CSH showed the same expression pattern for *ghr-1* and *ghr-2* as in the white muscle of the *in vivo* trial. However, in *in vitro* conditions, the action of the circulating GH is not present, and cells are only exposed to GH traces present within the fetal bovine serum supplementing the culture media, which in any case will affect equally to control and CSH incubated myocytes. Therefore, CSH by itself also seems to modulate the GH sensing in myocytes by an unknown mechanism. With respect to the IGF family, we observed an increase in the *igf-1b* and the *igf-2* relative expression after CSH exposure. Similar *igf-1* and *igf-2* induction was observed in Atlantic salmon (*Salmo salar*) and gilthead sea bream cultured myocytes after the exposure to nutrients, mainly amino acids (15, 70, 79). This would have sense since CSH is a natural precursor of taurine, a semi essential amino acid in carnivorous fish. Thus, these results suggest that together with the stimulatory effects on GH/IGF axis, CSH can have a direct effect on GH sensing and IGF-2 expression of muscle cells (14, 17). The modulation of the digestive function by the CSH and the possible improved entrance of nutrients would be an important factor that boosted the GH/IGF axis (45, 62, 80).

The IGFBPs and the IGFR regulate the availability and activity of the IGFs in the different tissues (14, 81). In the CSH-1.65 group there was a decrease in *igfbp-2a* in liver, which is the main IGF carrier in teleost (81). In early-stage zebrafish (*Danio rerio*), overexpression of *igfbp-2a* and *igfbp-2b* caused a reduction in body growth and developmental rate (82), suggesting a growth inhibitory role. In this sense, the hepatic downregulation of this binding protein in the animals fed with the CSH-1.65 of our study would be in line with the highest IGF-1 plasma levels observed in this group. However, variations among studies suggest a complex role of this binding protein in teleost growth, subjected to the physiological and species-specific context (81).

Furthermore, we observed a downregulation of the *igf-1ra* in white muscle and the *igf-1ra* and *igf-1rb* in stomach in fish fed with CSH-1.65. These reductions could be due to the negative feedback provoked by the boosted endocrine and paracrine function represented by the higher levels of IGF-1 in plasma and the relative expression of the different IGF-1 splice variants, respectively. Azizi et al. (2016) (15) found similar results in sea bream cultured myocytes incubated with amino acids, with the increased *igf-1* and *igf-2* previously mentioned and a reduction of the *igf1-ra* and *igf1-rb* receptors. However, in our *in vitro* experiment we found an upregulation of the *igf1-rb* instead its decrease, suggesting that the point of negative feedback had not yet been reached after 96 h of treatment.

## 5 Conclusions

Here we have demonstrated that the inclusion of CSH at 1.65 g/Kg and 3.3 g/Kg improved the hormonal balance of the somatotropic axis at both systemic and tissue levels. The upregulation of markers usually associated with an anabolic state such as GHR-1, IGF-1 and IGF-2 and the modulation of the IGFR and IGFBP in liver, white muscle and stomach, resulted altogether in an enhanced somatic growth. This condition would be mediated by the direct action on muscle, promoting the paracrine secretion and sensing of GH/IGF axis, but the effect on hypothalamic and intestinal somatostatin and the improvement in digestive absorption cannot be ignored and deserves future investigation. Thus, the use of CSH as a feed additive in a dose adjusted to the fish species and growing stage could be a very interesting strategy in Aquaculture.

## Data availability statement

The datasets presented in this study can be found in online repositories. The names of the repository/repositories and accession number(s) can be found in the article/**Supplementary Material**.

## Ethics statement

The animal study was reviewed and approved by Ethics and Animal Care Committee of the University of Barcelona.

## Author contributions

AS-M, JG, JB and JF-B conceptualized the study. RF provided the fish feed. AS-M performed the *in vivo* trial. AS-M, SB-P, EV, MP-A, IG-M, JF-B, JB and JG performed the sampling. AS-M and SB-P performed the *in vitro* trial. AS-M, SB-P, EV, MP-A, JC-G and JP-S performed the laboratory and data analyses. AS-M wrote the original draft. SB-P, EV, RF, JC-G, JP-S, JF-B, JB and JG critically reviewed the manuscript. JG, JB and JF-B acquired funding and administrated the project. All authors read and approved the final paper.
