# Supplementary material for: Cysteamine improves growth and the GH/IGF axis in gilthead sea bream (Sparus aurata): in vivo and in vitro approaches
Source: Front Endocrinol (Lausanne). 2023 Jul 20;14:1211470. doi: 10.3389/fendo.2023.1211470 (PMC10400459; doi:10.3389/fendo.2023.1211470)
Supplement: Supplementary file 1 [file Table_1.docx]

**Supplementary material**

**Table S1**

Primers used in the qPCR analyses. F: forward; R: reverse; Ta: annealing temperature.

| Type | Gene | Primer Sequences (5’-3’) | Ta (ºC) | Accession Number |
| --- | --- | --- | --- | --- |
| Housekeeping | *rpl27a* | **F:** AAGAGGAACACAACTCACTGCCCCAC  **R:** GCTTGCCTTTGCCCAGAACTTTGTAG | 68 | AY188520 |
|  | *rps18* | **F:** GGGTGTTGGCAGACGTTAC  **R:** CTTCTGCCTGTTGAGGAACCA | 60 | AM490061.1 |
|  | *ef1α* | **F:** CTTCAACGCTCAGGTCATCAT  **R:** GCACAGCGAAACGACCAAGGGGA | 60 | AF184170 |
|  | *Β-actin* | **F:** TCCTGCGGAATCCATGAGA  **R:** GACGTCGCACTTCATGATGCT | 60 | X89920 |
| GH-IGFs axis | *ghr-1* | **F:** ACCTGTCAGCCACCACATGA  **R:** TCGTGCAGATCTGGGTCGTA | 60 | AF438176 |
|  | *ghr-2* | **F:** GAGTGAACCCGGCCTGACAG  **R:** GCGGTGGTATCTGATTCATGGT | 60 | AY573601 |
|  | *igf-1a* | **F:** AGGACAGCACAGCAGCCAGACAAGAC  **R:** TTCGGACCATTGTTAGCCTCCTCTCTG | 60 | AY996779 |
|  | *igf-1b* | **F:** AGTCATTCATCCTTCAAGGAAGTGCATCC  **R:** TTCGGACCATTGTTAGCCTCCTCTCTG | 60 | EF688015 |
|  | *igf-1c* | **F:** ACAGAATGTAGGGACGGAGCGAATGGAC  **R:** TTCGGACCATTGTTAGCCTCCTCTCTG | 60 | EF688016 |
|  | *igf-2* | **F:** TGGGATCGTAGAGGAGTGTTGT  **R:** CTGTAGAGAGGTGGCCGACA | 60 | AY996778 |
|  | *igf-1ra* | **F:** AGCATCAAAGACGAACTGG  **R:** CTCCTCGCTGTAGAAGAAGC | 55 | KT156846 |
|  | *igf-1rb* | **F:** GCTAATGCGAATGTGTTGG  **R:** CGTCCTTTATGCTGCTGATG | 55 | KT156847 |
|  | *igfbp-1a* | **F:** AGTGCGAGTCCTCTCTGGAT  **R:** TCTCTTTAAGGGCACTCGGC | 60 | KM522771 |
|  | *igfbp-2a* | **F:** CGGGCTGCTGCTGACATACG  **R:** GTCCCGTCGCACCTCATTTG | 60 | AF377998 |
|  | *igfbp-4* | **F:** TCCACAAACCAGAGAAGCAA  **R:** GGGTATGGGGATTGTGAAGA | 60 | F5T95CD02JMZ9K |
